# Association between *CASR* Polymorphisms, Calcium Intake, and Colorectal Cancer Risk

**DOI:** 10.1371/journal.pone.0059628

**Published:** 2013-03-28

**Authors:** Kyee-Zu Kim, Aesun Shin, Jeongseon Kim, Ji Won Park, Sung Chan Park, Hyo Seong Choi, Hee Jin Chang, Dae Yong Kim, Jae Hwan Oh

**Affiliations:** 1 Molecular Epidemiology Branch, Research Institute, National Cancer Center, Goyang-si, Republic of Korea; 2 Center for Colorectal Cancer, National Cancer Center Hospital, National Cancer Center, Goyang-si, Republic of Korea; Ohio State University Medical Center, United States of America

## Abstract

**Aim:**

The current study aimed to assess the effect of dietary calcium intake and possible interactions with *calcium-sensing receptor* (*CASR)* gene polymorphisms on colorectal cancer risk.

**Methods:**

A total of 420 colorectal cancer cases and 815 controls were included in the analysis. Calcium intake was investigated using a 103 item semi-quantitative food frequency questionnaire, and four single nucleotide polymorphisms (SNPs) within the *CASR*, *rs10934578*, *rs12485716*, *rs2270916*, and *rs4678174*, were evaluated.

**Results:**

No SNPs were associated with colorectal cancer risk after adjusting for covariates. Overall, no significant effect modification by *CASR* polymorphisms on the association between calcium intake and colorectal cancer risk were detected. However, all 4 of the polymorphisms within the *CASR* showed significantly higher odds ratios for association with colorectal cancer risk in the low-calcium-intake group compared to the high-calcium-intake group. In the case of rs2270916, individuals with the CC genotype and low calcium intake showed an increased colorectal cancer risk compared to their counterparts with the TT genotype and high calcium intake (OR = 2.11, 95% CI = 1.27–3.51).

**Conclusions:**

Subjects with lower calcium intake exhibited a higher colorectal cancer risk compared with subjects with the same genotype who had higher calcium intake. Our results suggest that individuals who have low dietary calcium intake should be aware of their increased colorectal cancer risk and prevention strategies.

## Introduction

Colorectal cancer is one of the most common cancers worldwide [1], [2]. The incidence of colorectal cancer has increased in most Asian countries, including Korea [3].

A number of risk factors are associated with colorectal cancer, including dietary factors [4]. Previous studies have evaluated the associations between daily calcium or dairy food intake and colorectal cancer risk [5], [6], [7], [8], [9], [10], [11]. The results of those studies were inconsistent; however, a pooled analysis of 10 cohort studies [6] and a meta-analysis of 60 observational studies [10] consistently demonstrated positive associations between high calcium intake and reduced colorectal cancer risk.

A number of previous studies focused on the effects of genetic polymorphisms related to nutrition metabolism, such as *methylenetetrahydrofolate reductase* (*MTHFR*) [12], [13], *methionine synthase* (*MTR*) [14], [15], and *vitamin D receptor* (*VDR*) gene variants [16], [17], [18], [19], [20], [21]. The *MTHFR* 677TT and 1298CC polymorphisms seem to be associated with a reduced risk of CRC [12], [13]. Additionally, a tagSNP in the *MTR* (rs4659744) was significantly associated with reduced colorectal cancer risk [14]. Recently, the effects of the *calcium-sensing receptor* (*CASR)* gene on colorectal cancer have also garnered attention [22], [23], [24], [25]. Previous extracellular experiments have reported that *CASR* expression is involved in colorectal carcinoma differentiation through promoting E-cadherin expression and suppressing beta-catenin/TCF activation. [26], [27]. Several studies have also evaluated gene-environment (*CASR* and calcium intake) interactions associated with colorectal cancer risk [22], [25]. However, the role of *CASR* and calcium intake in colorectal cancer incidence is not fully understood due to the relatively small sample sizes and inconclusive results of these studies.

This case-control study aimed to explore the associations between *CASR* variants with daily calcium intake and colorectal cancer risk in Korea. We examined the main effects of the *CASR* on colorectal cancer by sex and cancer sites. Then, we evaluated possible gene-environment interactions between *CASR* polymorphisms and daily calcium intake in the context of colorectal cancer risk.

## Materials and Methods

### Study Participants and Data Collection

All participants provided written informed consent to participation, and the study protocol was approved by the Institutional Review Board of the National Cancer Center. Eligible colorectal cancer patients were recruited from the Center for Colorectal Cancer, National Cancer Center in Korea from August 2010 to December 2011. Among 702 eligible colorectal cancer patients, 671 patients were contacted, and 554 agreed to participate in the study. Patients from whom blood samples were not obtained or who did not complete a structured questionnaire were excluded from the analysis. As a result, 420 colorectal cancer patients remained in the final analysis. Eligible controls were recruited from March 2010 to August 2011 from among patients who visited the Center for Early Detection and Prevention of the National Cancer Center, Korea, for regular health examination. Among 4,514 eligible controls, 3,687 agreed to participate in the study; 1,014 participants who did not complete a self-administered questionnaire were excluded. The remaining 2,673 participants were 1∶2 matched with 420 colorectal cancer patients by sex and 5-year age range, and 960 subjects were selected. Among these 960 controls, 145 whose blood samples were not obtained were excluded. Thus, information from 1,235 participants (420 cases and 815 controls) was considered in the final analysis. Information on total energy and calcium intake were available from 1,188 of these subjects for inclusion in the analyses to evaluate the joint effects of calcium intake and *CASR* polymorphisms on colorectal cancer.

Information on subject age, education level, smoking and alcohol drinking habits, household income, menopausal status (women only) and history of hormone replacement therapy (post-menopausal women only) were obtained by questionnaire. The cancer site was classified as proximal colon, distal colon, or rectum for each patient. Subject height and weight were measured, and body mass index (BMI) was calculated.

The regular dietary intake of each study participant was recorded using a semi-quantitative food frequency questionnaire (SQFFQ). The reliability and validity of the FFQ was demonstrated in a previous report [28]. The FFQ consisted of 103 food items, and participants were asked to report the average frequencies and portion sizes of the foods that they ate during the previous year. For each of the 103 food items, nutrient quantity per 100 g was measured and converted to daily nutrient intake.

### *CASR* Genotyping (TaqMan Assay)

Based on the literature, four SNPs of *CASR* that showed an association with colorectal cancer risk were selected for analysis [22], [24]. The genotyping of *CASR* polymorphism (rs10934578, rs12485716, rs2270916, rs4678174) was screened using the TaqMan fluorogenic 5′ nuclease assay (ABI, Foster City, CA, USA). The polymerase chain reaction (PCR) was performed in a 5 µl reaction containing 10 ng genomic DNA, 2.5 µl TaqMan Universal PCR Master Mix, and 0.13 µl of 20X Assay Mix (Assay ID C___2684958_10). The thermal cycling conditions were as follows: 50°C for 2 min to activate the uracil N-glycosylase and to prevent carry-over contamination, 95°C for 10 min to activate the DNA polymerase, followed by 45 cycles of 95°C for 15 s and 60°C for 1 min. All PCR assays were performed in 384-well plates in a Dual 384-Well GeneAmp PCR System 9700 (ABI, Foster City, CA, USA), and the endpoint fluorescent readings were taken by an ABI PRISM 7900 HT Sequence Detection System (ABI, Foster City, CA, USA). Duplicate samples and negative controls were included to ensure genotyping accuracy.

### Statistical Analysis

To compare general characteristics between cases and controls, chi-square tests and Student’s t-tests were performed. Chi-square tests were employed to explore the distributions of each genotype between the calcium intake groups. First, we performed separate chi-square analyses for each calcium intake group (low intake group and high intake group). The cut-off point for median calcium intake was based on the distribution in the control group. Participants whose calcium intake fell below the median value were classified in the low calcium intake group; the remaining participants were classified in the high calcium intake group. Then, Mantel-Haenszel chi-square tests were used to evaluate combined statistics. We investigated the main effects of *CASR* on colorectal cancer according to genetic inheritance models, including additive, dominant, and recessive models. To investigate the association between *CASR* polymorphisms and colorectal cancer risk by cancer site, a polytomous logistic regression model was used. We used logistic regression models to calculate odds ratios for colorectal cancer risk. Interaction p-values were calculated using the likelihood ratios from the chi-square distribution function. SAS version 9.1 (SAS Institute Inc., Cary, NC) was used for all analyses.

## Results

The basic characteristics and demographic descriptions of the study participants are presented in Table 1. Among the male participants, differences in age, height, weight, BMI, alcohol drinking status, education, and household income were observed between colorectal cancer patients and controls. In female subjects, differences in height, BMI, smoking status, alcohol status, education level, and household income were observed between patients with different colorectal cancer status. It also appeared that among the post-menopausal women, colorectal cancer patients took more HRT than did control subjects (p = 0.019, data not shown).

**Table 1:** Study participant characteristics, mean (standard deviation).

		Male		p	Female		p
		case (n = 282)	control (n = 515)		case (n = 138)	control (n = 300)	
Age groups, N (%)	30∼39	11 (3.9)	18 (3.5)	<0.001	9 (6.5)	26 (8.7)	0.391
	40∼49	52 (18.4)	119 (23.1)		22 (15.9)	48 (16.0)	
	50∼59	90 (31.9)	219 (42.5)		50 (36.2)	126 (42.0)	
	60∼	129 (45.7)	159 (30.9)		57 (41.3)	100 (33.3)	
Height (cm)		168.1 (6.1)	169.8 (5.9)	<0.001	155.3 (6.1)	157.6 (4.7)	<0.001
Weight (kg)		66.6 (9.7)	70.4 (10.6)	<0.001	57.0 (8.7)	57.6 (8.7)	0.450
BMI (kg/m2)		23.6 (2.9)	24.3 (2.7)	<0.001	24.3 (3.7)	23.1 (2.5)	0.061
Smoking status,	never	65 (23.2)	104 (20.2)	0.304	123 (89.1)	293 (98.0)	0.001
N (%)	former	130 (46.3)	267 (52.0)		5 (3.6)	4 (1.3)	
	current	86 (30.6)	143 (27.8)		10 (7.3)	2 (0.7)	
Alcohol drinking,	never	48 (17.1)	85 (16.5)	0.002	82 (59.4)	189 (63.2)	0.024
N (%)	former	64 (22.8)	69 (13.4)		21 (15.2)	21 (7.0)	
	current	169 (60.1)	360 (70.0)		35 (25.4)	89 (30.0)	
Education,	elementry school or less	40 (14.2)	27 (5.5)	<0.001	51 (37.0)	24 (8.6)	<0.001
N (%)	middle school	52 (18.5)	41 (8.4)		25 (18.1)	24 (8.6)	
	high school	98 (34.9)	156 (31.8)		39 (28.3)	142 (50.7)	
	collge or more	91 (32.4)	266 (54.3)		23 (16.7)	90 (32.1)	
Monthly income	∼100	44 (15.7)	24 (5.3)	<0.001	33 (24.0)	16 (6.4)	<0.001
(Won), N (%)	100–200	71 (25.3)	70 (15.4)		22 (15.9)	42 (16.8)	
	200–400	101 (35.9)	210 (46.3)		53 (38.4)	102 (40.8)	
	400∼	65 (23.1)	150 (33.0)		30 (21.7)	90 (36.0)	
Post-menopausal, N (%)					101 (73.2)	212 (70.7)	0.587
HRT (post-menopausal women only), N (%)	never				4 (4.0)	20 (9.8)	0.019
	former				14 (14.0)	48 (23.4)	
	current				82 (82.0)	137 (66.8)	
Total energy intake (kcal/day)		2131.5 (484.1)	1790.9 (565.5)	<0.001	1805.0 (518.9)	1709.9 (626.1)	0.112
Total calcium intake (mg/day)		438.8 (178.0)	440.3 (243.4)	0.922	444.2 (210.5)	543.9(360.8)	0.001
Energy adjusted total calcium intake (mg/day)		382.6 (128.9)	451.3 (204.0)	<0.001	452.9 (169.7)	572.2(251.5)	<0.001

Table 1 also shows the subjects’ total energy intake, total calcium intake, and energy-adjusted total calcium intake. Among all subjects, the total energy intake was higher for colorectal cancer patients, whereas energy-adjusted total calcium intake was higher for the control group.

All 4 SNPs were in Hardy-Weinberg equilibrium and consistent with the genotype distribution in the Asian population in HapMap. The association between *CASR* polymorphisms and colorectal cancer are presented in Table 2, subdivided according to genetic inheritance mode. No SNPs showed significant effects on colorectal cancer after adjusting for age, sex, smoking status, alcohol drinking status, and monthly household income. Similarly, we found no associations between *CASR* variants and colorectal cancer site, i.e., proximal colon, distal colon, and rectum (Table 3).

**Table 2:** The associations between *CASR* polymorphisms and colorectal cancer risk.

Genetic inheritance mode	SNPs (n cases/n controls)		OR^a^	95% CI
Additive	rs10934578	TT (85/158)		
		GT (203/385)	1.13	0.84–1.52
		GG (124/254)	1.12	0.78–1.62
	rs12485716	GG (80/149)		
		GA (199/396)	1.03	0.78–1.38
		AA (135/266)	1.09	0.76–1.58
	rs2270916	TT (77/132)		
		CT (192/389)	0.98	0.74–1.31
		CC (149/291)	1.14	0.79–1.65
	rs4678174	TT (87/160)		
		CT (202/387)	1.18	0.88–1.58
		CC (122/260)	1.17	0.82–1.69
Dominant	rs10934578	GG (85/158)		
		TT+GT (327/639)	1.04	0.76–1.43
	rs12485716	AA (80/149)		
		GG+GA (334/662)	1.07	0.77–1.48
	rs2270916	CC (77/132)		
		TT+CT (341/680)	1.15	0.83–1.61
	rs4678174	CC (87/160)		
		TT+CT (324/647)	1.07	0.78–1.46
Recessive	rs10934578	TT (124/254)		
		GG+GT (288/543)	0.89	0.67–1.17
	rs12485716	GG (135/266)		
		AA+GA (279/545)	0.95	0.73–1.25
	rs2270916	TT (149/291)		
		CC+CT (269/521)	0.98	0.75–1.27
	rs4678174	TT (122/260)		
		CC+CT (289/547)	0.66	0.44–1.01

Abbreviations: SNP = single nucleotide polymorphism; OR = odds ratio; CI = confidence interval.

a Odds ratios adjusted for age (categorical), sex, smoking, alcohol, and monthly household income.

**Table 3:** The associations between *CASR* polymorphisms and colorectal cancer risk by cancer site.

Genetic inheritance mode	SNPs		Proximal colon		Distal colon		Rectum	
			OR^a^	95% CI	OR^a^	95% CI	OR^a^	95% CI
Additive	rs10934578	TT						
		GT	1.31	0.70–2.44	1.30	0.83–2.03	0.75	0.51–1.09
		GG	0.96	0.42–2.18	1.30	0.76–2.24	0.76	0.47–1.23
	rs12485716	GG						
		GA	1.10	0.61–1.99	1.22	0.79–1.90	0.71	0.49–1.03
		AA	1.00	0.46–2.16	1.35	0.79–2.32	0.74	0.46–1.20
	rs2270916	TT						
		CT	1.07	0.60–1.91	1.12	0.73–1.71	0.75	0.52–1.09
		CC	1.00	0.46–2.19	1.36	0.80–2.31	0.87	0.54–1.41
	rs4678174	TT						
		CT	1.38	0.73–2.62	1.29	0.82–2.00	0.81	0.55–1.18
		CC	1.13	0.51–2.54	1.23	0.72–2.12	0.80	0.50–1.29
Dominant	rs10934578	GG						
		TT+GT	0.81	0.40–1.63	1.10	0.70–1.73	0.90	0.59–1.38
	rs12485716	AA						
		GG+GA	0.94	0.48–1.86	1.19	0.75–1.89	0.89	0.58–1.39
	rs2270916	CC						
		TT+CT	0.96	0.47–1.95	1.27	0.80–2.03	1.02	0.65–1.58
	rs4678174	CC						
		TT+CT	0.92	0.47–1.82	1.05	0.67–1.66	0.91	0.60–1.39
Recessive	rs10934578	TT						
		GG+GT	0.83	0.46–1.51	0.77	0.50–1.17	1.33	0.94–1.89
	rs12485716	GG						
		AA+GA	0.93	0.53–1.64	0.79	0.52–1.20	1.39	0.98–1.97
	rs2270916	TT						
		CC+CT	0.95	0.55–1.65	0.85	0.57–1.26	1.28	0.91–1.80
	rs4678174	TT						
		CC+CT	0.76	0.42–1.41	0.79	0.52–1.20	1.24	0.87–1.76

Abbreviations: SNP = single nucleotide polymorphism; OR = odds ratio; CI = confidence interval.

a Odds ratios were obtained from polytomous regression model; adjusted for age (categorical), sex, smoking, alcohol, and monthly household income.

Table 4 shows the interaction effects between *CASR* genotypes and calcium intake. We did not find any overall interaction effects in our analyses. However, all 4 of the *CASR* polymorphisms investigated showed significant odds ratios for association with colorectal cancer risk in the low calcium intake group compared with the high calcium intake group after adjusting for age, sex, smoking status, alcohol drinking, and monthly household income. For rs2270916, participants who had the CC genotype and low calcium intake showed the highest colorectal cancer risk among all subjects (OR = 2.11, 95% CI = 1.27–3.51), demonstrating an increased risk in comparison to the high calcium intake group with the same genotype.

**Table 4:** The interaction effects between energy-adjusted daily calcium intake and *CASR* polymorphisms.

SNP		OR^a^ (95% CI)		p for interaction
		low intake^b^	high intake^c^	
rs10934578	TT	1.77 (1.14–2.77)	1	0.295
	GT	1.92 (1.28–2.89)	0.99 (0.65–1.51)	
	GG	1.84 (1.12–3.00)	0.88 (0.50–1.56)	
rs12485716	GG	1.90 (1.23–2.95)	1	0.769
	GA	1.72 (1.15–3.58)	0.94 (0.62–1.44)	
	AA	1.89 (1.14–3.11)	0.85 (0.47–1.61)	
rs2270916	TT	2.01 (1.31–3.09)	1	0.375
	CT	1.83 (1.22–2.75)	0.97 (0.63–1.48)	
	CC	2.11 (1.27–3.51)	1.02 (0.57–1.83)	
rs4678174	TT	1.64 (1.05–2.56)	1	0.883
	CT	1.83 (1.22–2.76)	0.95 (0.62–1.44)	
	CC	1.73 (1.06–2.83)	0.84 (0.48–1.49)	

Abbreviations: SNP = single nucleotide polymorphism; OR = odds ratio.

a Adjusted for age (categorical), sex, smoking, alcohol, and monthly household income.

b, c criteria for low and high calcium intakes was 417.06 mg per a day.

## Discussion

In this study, we aimed to explore the effects of *CASR* polymorphisms and calcium intake on colorectal cancer. To our knowledge, few studies have focused on gene-environment interactions between *CASR* variants and dietary calcium intake with respect to colorectal cancer. This is the first study that demonstrates the effects of *CASR* gene polymorphisms and *CASR*-calcium intake interactions in a Korean population with a substantial number of subjects.

A number of previous studies have proposed that calcium may protect against colorectal cancer risk by binding fatty acids and secondary bile acids in the colonic lumen [29], [30], [31]. This activity can protect epithelial cells from mutagens in the colon. In addition, calcium is involved in intracellular mechanisms that affect cell proliferation and differentiation [32], [33], and calcium decreases epithelial cell proliferation directly [34].

*CASR* has been implicated in mediating the anticarcinogenic effects of calcium on colorectal cancer [26], [27], [35], [36]. *CASR* expression is detected in both the basolateral and luminal surfaces of the colon, implying that this gene is involved in the pathway that regulates the calcium concentration in the colon and in the blood [36], [37], [38]. According to a review describing the *CASR* molecular pathway in colorectal cancer [36], calcium activates certain signalling pathways that participate in cell growth and differentiation via *CASR*, including the promotion of E-cadherin expression and the suppression of β-catenin and T-cell factor activation, as well as the activation of the p38 mitogen-activated protein kinase cascade [39]. In addition, laboratory results suggested that CASR might mediate the pro-cell proliferative effects of low intestinal calcium concentration [26], [35].

The results of the current study showed that *CASR* polymorphisms themselves were not likely to be correlated with colorectal cancer. This is consistent with previous observational studies that demonstrated no association between *CASR* polymorphisms and colorectal cancer [22], [25], although several studies have reported significant associations between SNPs in *CASR* and colorectal cancer risk [23], [24], [40]. The SNPs that were included in this study, *rs10934578*, *rs12485716*, *rs2270916*, and *rs4678174*, were not associated with overall colorectal cancer in previous studies, although *rs12485716* and *rs2270916* were associated with proximal colon cancer risk [22]. These associations with proximal colon cancer were not replicated in our study. *Rs4678174* was demonstrated to have no relationship with colorectal cancer in two studies that were performed in European [25], North American, and Australia [24] populations, respectively. This study could serve as further evidence from an Asian population.

We did not find any overall interaction effects between *CASR* polymorphisms and daily calcium intake on colorectal cancer. However, among patients with the same *CASR* genotypes, subjects with lower calcium intake showed higher colorectal cancer risk than did subjects with higher calcium intake. To date, no major studies have demonstrated interaction effects between calcium intake and *CASR* polymorphisms on colorectal cancer. Previous studies that analysed interaction effects between calcium intake and *CASR* polymorphisms noted no associations with colorectal cancer [22], [25]. Our results are generally consistent with those null associations; however, we noticed a meaningful increase in colorectal cancer risk in the low calcium intake group compared to the high calcium group after stratification by *CASR* risk genotype. Though further study is required to fully understand how dietary calcium and *CASR* interact to modulate colorectal cancer carcinogenesis, our observations suggest that individuals who have low dietary calcium intake should be aware of their colorectal cancer risk and prevention strategies.

Our study has several limitations. Because we used cancer examination screenees as controls, they might be healthier than the general population, although they were recruited from the hospital. In addition, to minimize the potential information bias between groups, the interviewer who was in charge of data collection from the food frequency questionnaire for cases was also involved in the survey for controls. We included only 4 SNPs within the *CASR* in our investigation of the gene-only and gene-environment interaction effects on colorectal cancer. To evaluate the effects of *CASR* polymorphism and calcium intake on colorectal cancer, further studies which include more SNPs on *CASR* would be required. The criterion used for calcium intake is another limitation of our study. Grouping of calcium intake was based on average intake value of study population, not recommended dietary allowances (RDA) for Korean population. However, because our calcium intake criterion was lower than the RDA [41], we could conclude that lower calcium intake was likely to be a risk factor for colorectal cancer.

In conclusion, we demonstrated that there is an increased risk of colorectal cancer in subjects with low calcium intake and *CASR* polymorphisms. Further investigations will be required to explain the genetic and gene-environmental effects on colorectal cancer in larger sample sizes and using additional *CASR* functional regions or variants. Increasing the calcium intake to the DRI level is recommended to prevent colorectal cancer in Korea.
